# Association of rare variation in the glutamate receptor gene *SLC1A2* with susceptibility to bipolar disorder and schizophrenia

**DOI:** 10.1038/ejhg.2014.261

**Published:** 2014-11-19

**Authors:** Alessia Fiorentino, Sally I Sharp, Andrew McQuillin

**Affiliations:** 1 Molecular Psychiatry Laboratory, Division of Psychiatry, Rockefeller Building, University College London, London, UK

## Abstract

The *SLC1A2* gene encodes the excitatory amino acid transporter 2 (EAAT2). Glutamate is the major mediator of excitatory neurotransmission and EAAT2 is responsible for clearing the neurotransmitter from the synaptic cleft. Genetic variation in *SLC1A2* has been implicated in a range of neurological and neuropsychiatric conditions including schizophrenia (SZ), autism and in core phenotypes of bipolar disorder (BD). The coding and putative regulatory regions of *SLC1A2* gene were screened for variants using high resolution melting or sequenced in 1099 or in 32 BD subjects. Thirty-two variants were detected in the *SLC1A2* gene. Fifteen potentially etiological variants were selected for genotyping in 1099 BD and 1095 control samples. Five amino acid changing variants were also genotyped in 630 participants suffering from SZ. None of the variants were found to be associated with BD or SZ or with the two diseases combined. However, two recurrent missense variants (rs145827578:G>A, p.(G6S); rs199599866:G>A, p.(R31Q)) and one recurrent 5′-untranslated region (UTR) variant (ss825678885:G>T) were detected in cases only. Combined analysis of the recurrent-case-only missense variants and of the case-only missense and 5′-UTR variants showed nominal evidence for association with the combined diseases (Fisher's *P*=0.019 and 0.0076). These findings are exploratory in nature and await replication in larger cohorts, however, they provide intriguing evidence that potentially functional rare variants in the *SLC1A2* gene may confer susceptibility to psychotic disorders.

## Introduction

Glutamate is considered to be the major mediator of excitatory neurotransmission in the central nervous system. At high extracellular concentrations, glutamate can induce neuronal cell death by a complex mechanism termed excitotoxicity.^1^ Extracellular glutamate is predominantly cleared by the solute carrier 1 (SLC1) family of proteins. This family of proteins comprises five high-affinity glutamate transporters (excitatory amino acid transporter 3 (EAAT3), EAAT2, EAAT1, EAAT4 and EAAT5) and two amino acid transporters (ASCT1 and AAAT). *SLC1A2* encodes EAAT2, which is also known as the glial high-affinity glutamate transporter GLT1 in the mouse. It has a crucial role in removing glutamate from the synaptic cleft and has been found to be responsible for about 90% of total glutamate uptake in most brain areas.^2, 3^ This process modulates the termination of glutamatergic synaptic signalling and prevents excitotoxic effects of glutamate on post-synaptic neurones. EAAT2 is predominantly expressed in astrocytes under normal conditions.^4^ It is also detected in oligodendrocytes and in neurons^4, 5, 6, 7^ throughout the spinal cord and the brain, particularly in the cerebral cortex and hippocampus.^8^ The *SLC1A2* gene consists of 11 protein-coding exons and spans more than 50 kb of genomic DNA on chromosome 11p13-12.^9^ Several post-transcriptionally regulated forms of EAAT2 have been identified. Alternative splicing of the 5′- and 3′-untranslated regions (UTRs) produces different mRNA isoforms^10, 11, 12, 13, 14^ as well as proteins with different amino- (N-) and carboxy- (C-) termini.^15, 16, 17^ The 3′-UTR has a variety of regulatory elements^14^ including enhancers, AU-rich elements, as well as putative polyadenylation sites. AU-rich elements define the cytoplasmatic half-life of mRNA molecules^18, 19^ and polyadenylation is essential for nuclear mRNA export, translation and control of mRNA decay.^20, 21^ Besides diverse N- and C-termini, exon-skipping splice variants of EAAT2 have been described in brain tissue samples and these include: an exon 5 and 6 partial deletion,^22^ an exon 7 deletion,^23^ an exon 8 deletion,^24^ an exon 9 deletion^25^ and an exon 7 and 9 deletion.^26^ Evidence of epigenetic regulation of human EAAT2 transcription based on methylation of the promoter has also been identified.^27, 28^

Dysregulation of EAAT2 is associated with several neurological diseases including amyotrophic lateral sclerosis,^29^ Alzheimer's disease, epilepsy, as well as several psychiatric diseases such as schizophrenia (SZ), depression^30^ and autism.^31, 32^ The *SLC1A2* SNP rs3794087 was found to be associated with essential tremors in a GWAS study.^33^ However, this finding has not been consistently replicated.^34, 35, 36, 37^

There is evidence which suggests that alterations in the glutamatergic systems may contribute to the pathophysiology of depression,^38^ with elevated levels of glutamate observed in the cerebral cortex of depressed patients.^39^ Glutamate signalling is involved in neurological mechanisms implicated in the etiology of bipolar disorder (BD) such as brain development and synaptic plasticity.^40^ In post-mortem brain tissue from BD patients, altered levels of glutamate/glutamine, N-methyl-D-aspartate receptors^41^ and *SLC1A2* protein and mRNA^42^ have been found. Reduced expression of the membrane transporter *SLC1A2* was also reported in major depression while a trend in the same direction has been observed in BD subjects.^43^ Differential expression of *SLC1A2* in the locus coeruleus has been reported in major depression but not in BD subjects.^44^ Two SNPs in *SLC1A2* have been reported to be associated with the core phenotypes of BD: rs4755404:C>G was reported to be associated with attempted suicide^45^ and rs4354668:A>C was reported to influence the total episode recurrence rate and the efficacy of lithium treatment response in a sample of Italian patients with BD.^46^ rs4354668:A>C is a functional polymorphism and was shown to significantly influence grey matter volume in BD subjects with low adverse childhood experiences, with the wild-type T homozygote presenting the lowest volume, the G homozygote reporting the highest volume and heterozygotes showing an intermediate phenotype.^47^ The same SNP has a similar effect in a group of SZ subjects: carriers of the G allele had lower grey matter volumes and worse working memory performance than T/T homozygotes.^48^ Significant dysregulation of *SLC1A2* expression in the dorsolateral prefrontal cortex in BD and SZ subjects compared with control individuals has been reported.^49^ Decreased EAAT2 protein levels were found in the superior temporal gyrus and in the hippocampus of SZ subjects^50^ and EAAT2 glycosylation was found to be altered in patients with SZ compared with controls.^51^ Allelic association between *SLC1A2* and BD was detected by our laboratory in the UCL-STEPBD genome-wide association study (GWAS).^52^ In the UCL sample 10 markers within the gene showed *P-*values ranging from 0.0011 to 0.038.

## Materials and methods

### Subjects

This study included 1099 affected BD research subjects and 630 subjects affected with SZ. Ancestry screening was used as a selection criterion for the inclusion of cases and screened controls. Samples were included if at least three out of four grandparents were English, Irish, Scottish or Welsh and if the fourth grandparent was non-Jewish European, before the EU enlargement in 2004. All SZ cases were selected for having prior International Classification of Diseases 10 (ICD10) diagnosis of SZ made by National Health Service (NHS) psychiatrists. The BD cases were sampled in two cohorts. The first cohort, UCL1, includes 506 Bipolar 1 (BP1) research subjects defined by the presence of mania and hospitalisation according to research diagnostic criteria (RDC).^53^ The second cohort, UCL2, consists of 593 subjects with BP1 or BP2. BD and SZ research subjects had been given a clinical diagnosis of ICD-10 BD or SZ and were needed to fulfil the criteria for the lifetime version of the SZ and Affective Disorder Schedule (SADS-L),^54^ which provides RDC.^53^ The sample of 1095 controls comprised 615 screened subjects who had no first degree family or personal history of psychiatric illness and an additional 480 unscreened normal British subjects obtained from the European Collection of Animal Cell Cultures (ECACC). NHS multicentre research ethics approval was obtained. All participants provided signed consent.

DNA samples were collected from blood samples from the UCL1, SZ and control cohorts, and from saliva samples from the UCL2 cohort. DNA from blood samples was extracted using standard phenol–chloroform method and from saliva samples using the Oragene protocol for DNA extraction (DNA Genotek, Ottowa, ON, Canada).

### Detection and evaluation of new variants

High resolution melting (HRM) variant screening was used to identify BD susceptibility variants in the coding exons, 5′-UTR, intron/exon junctions and the putative promoter region (500 bp from the 5′-UTR (chr11: 35441106-35441687 GRCh37/hg19) of the main isoform ENSP00000278379). HRM was performed using 18 primer pairs in 1099 BD cases. Reactions were carried out on a LightCycler 480 (Roche, Burgess Hill, UK). Primer sequences and reagents are shown in Supplementary Table 1. Samples with abnormal HRM curves were then sequenced using the Big Dye terminator v3.1 Cycle Sequencing kit (Applied Biosystems, Warrington, UK) on an ABI 3730*xl* DNA Analyzer (Applied Biosystems). Sequencing data was analysed using the Staden Package.^55^ Variants that were not previously reported were submitted to dbSNP (http://www.ncbi.nlm.nih.gov/SNP/). All variant identifiers are shown in Table 1.

Thirty-two BD cases were selected from among those cases who had inherited an *SLC1A2* haplotype of rs7105037, rs7105418, rs4756201, rs3794099, rs1042113, rs3818275, rs41352148, rs11033052, rs2281634, rs4756208, rs2421765 and rs3794089^52^ that was associated with BD. Samples were selected for sequencing on the basis of being carriers of the alleles 5′-GCCCTTACCAAA-3′ for the SNPs listed above. Sequencing was carried out on the putative promoter region as defined above, the 5′-UTR and the 3′-UTR (primers in Supplementary Table 2). Sequencing was performed as described above.

Non-synonymous variants, intron/exon junction variants and SNPs in the putative regulatory region, whose minor allele frequency was unknown or <0.01 in the 1000 genomes project (release 20110521),^56^ were considered for further analysis.

Bioinformatic analysis, to determine potentially functional SNPs, was carried out using the UCSC genome browser (http://genome.ucsc.edu/), TESS (http://www.cbil.upenn.edu/cgi-bin/tess/tess), PolyPhen2^57^ (http://genetics.bwh.harvard.edu/pph2/), SIFT (http://sift.jcvi.org/), Splice Site Prediction by Neural Network (http://www.fruitfly.org/seq_tools/splice.html), miRanda (http://www.microrna.org/microrna/home.do) and targetscan 6.2 (http://www.targetscan.org/).

### Genotyping

Genotyping of the selected SNPs in 1099 BD cases and 1095 ancestrally matched controls was performed in-house with allele-specific PCR using KASPar reagents (LGC Genomics, Hoddesdon, UK) on a LightCycler 480 (Roche) real-time PCR machine. The non-synonymous variants were also genotyped in 630 SZ samples. For all SNPs genotyped, 17% of samples were duplicated to detect error and confirm reproducibility of genotypes. Allele-specific primers were designed for each of the SNPs using Primer Picker (LGC Genomics) as shown in Supplementary Table 3. All these data were analysed to confirm Hardy–Weinberg equilibrium (HWE). Allelic association analysis of single variants was performed using PLINK (v1.07)^58^ and these were supplemented with Fisher's exact tests when cell frequencies were <10. The data for recurrent-case-only variants were combined and tested for association with BD and SZ against the control subjects using a Fisher's exact test. Significance values shown for all analyses are uncorrected for multiple testing and a cut-off significance value of *P*<0.05 was used.

## Results

### Variant selection

A total of 32 SNPs were detected by HRM analysis across the putative promoter region, 5′-UTR, exons, intron/exon junctions of *SLC1A2* and a further 8 SNPs were detected by sequencing analysis of 5′- and 3′-UTR regions (Table 1). These included five synonymous coding base pair changes, ss825678894:C>T, rs752949:G>A, rs1042113:A>G, rs16927239:C>T and rs139804773:C>T; five non-synonymous coding base pair changes, rs145827578:G>A, ss825678885:G>T, rs199599866:G>A, ss825678893:C>T and ss825678895:C>T; ten SNPs in introns, rs5791053:->T, rs55643101:T>G, ss825678886:A>G, rs2273687:C>G, rs2273686:C>T, rs56028027:A>G, rs3895234:T>C, rs116776036:C>T, rs2273689:T>C and ss825678896:G>T; one variant in intron/exon junctions, rs56205617:G>A; seven SNPs in the 5′-UTR, rs111885243:C>A, rs4354668:A>C, rs116392274:C>A, ss825678882:A>G, ss825678883:C>G, ss825678884:G>A, rs1885345:T>C; nine SNPs in the 3′-UTR, rs1570216:A>G, rs10742339:T>A, rs147294857:C>T, rs10768121:T>G, ss825678897:A>G, rs12361171:A>T, rs3812779:C>T, rs1043101:T>C, rs3088168:A>G; and three putative promoter SNPs, rs77619780:G>A, ss825678881:G>C and rs11033118:C>T. Non-synonymous variant ss825678885:G>T in the uc021qfy.1 isoform is also a 5′-UTR variant in the ENSP00000379099 isoform.

Non-synonymous variants, intron/exon junction variants and SNPs in the putative regulatory region, whose minor allele frequency was unknown or <0.01, were considered for further analysis.

After filtering, 15 variants remained and these comprised 6 known and 9 unknown variants. Two known and one previously unknown variant were present in the putative promoter region (rs77619780:G>A, ss825678881:G>C and rs11033118:C>T), three previously unknown variants were in the 5′-UTR (ss825678882:A>G, ss825678883:C>G and ss825678884:G>A), two known and three previously unknown SNPs were non-synonymous (rs145827578:G>A, ss825678885:G>T, rs199599866:G>A, ss825678893:C>T and ss825678895:C>T), one known variant was in an intron/exon junction (rs56205617:G>A) and two known variants and one previously unknown variant were in the 3′-UTR (rs147294857:C>T, ss825678897:A>G and rs3812779:C>T).

Bioinformatics analysis of putative promoter and 5′-UTR region SNPs for altered transcription factor binding indicated that the mutant alleles were more likely to bind to a decreased range of transcription factors compared with their respective common alleles. rs77619780:G>A was predicted to destroy a binding site for three transcription factors (CCAAT/enhancer-binding protein alpha, Upstream stimulatory factor, Specificity protein 1 (Sp1)), ss825678881:G>C was predicted to destroy a binding site for one transcription factor (Sp1) and ss825678883:C>G was predicted to destroy a binding site for two transcription factors (Early growth response protein 1 and Wilms tumor protein). rs11033118:C>T and ss825678884:G>A were not predicted to alter transcription factor binding sites, while ss825678882:A>G was predicted to create a binding site for the Sp1 transcription factor.

rs145827578:G>A causes a non-conservative amino acid change from glycine to serine at position 6 (p.(G6S)) of the main isoform. The p.(G6S) amino acid substitution was predicted to be ‘benign' with a score of 0.439 (sensitivity: 0.89 and specificity: 0.90) by PolyPhen-2 and ‘tolerated' with a score of 0.056 by SIFT. The same substitution in the ENSP00000476124 isoform is predicted to be ‘possibly damaging' with a score of 0.575 (sensitivity: 0.88 and specificity: 0.91) by Polyphen2 (Table 2). In this isoform, 11 amino acids substitute the last 22 amino acids of the ENSP00000278379 isoform.

rs199599866:G>A causes a non-conservative amino acid change from arginine to glutamine at position 31 (p.(R31Q)) of the main isoform. The p.(R31Q) amino acid substitution was predicted to be ‘benign' with a score between 0 and 0.003 depending on the isoform (sensitivity: 0.99; specificity: 0.30) by PolyPhen-2, and ‘tolerated' with a score between 0.191 and 0.215 depending on the isoform by SIFT.

The variant ss825678893:C>T causes an alanine to valine substitution in position 7 (p.(A7V)) of the main isoform and the SNP ss825678895:C>T causes a non-conservative amino acid change from arginine to cysteine in position 572 (p.(R572C)) of the main isoform. The two new non-synonymous variants were predicted to be ‘probably damaging' by PolyPhen2 and ‘damaging' by SIFT in the main isoform.

ss825678885:G>T causes a substitution from alanine to serine at position 20 (p.(A20S)) of the alternative isoform uc021qfy.1. This isoform is a non-RefSeq transcript and it was not possible to predict the effect that the variant may have on protein structure or function using Polyphen2 or SIFT. In the ENSP00000379099 isoform the variant is in the 5′-UTR and was predicted to create a binding site for three transcription factors (estrogen receptors, Chicken Ovalbumin Upstream Promoter transcription factor and Sp1).

The intron/exon junction variant rs56205617:G>A was predicted to destroy the donor site of the second exon ENSE00003694146 of the isoform ENSP00000379099 by the BDGP splice site predictor programme.

Of the three variants in the 3′-UTR, only the previously unreported SNP ss825678897:A>G was predicted to alter the binding site for two microRNA species— miR-664 and miR-664b.

### Genotyping

Assays were designed for 15 SNPs, which passed filtering tests for genotyping in the complete UCL BD case-control sample. Five non-synonymous SNPs were also genotypes in 630 SCZ samples. rs145827578:G>A was detected in three BD and two SZ samples and not in any of the control subjects. These data were not significantly associated with either disease on their own or in a combined analysis. None of the others variants were found to be associated with BD, as shown in Table 3.

When the data for case-only recurrent missense variants rs145827578:G>A (p.(G6S)) and rs199599866:G>A (p.(R31Q)) in BD and SZ were combined in a burden test there was evidence for association with the two diseases (Fisher's *P*=0.0458; see Table 4). In a further analysis the data from a third case-only variant ss825678885:G>T (5′-UTR of uc021qfy.1 isoform and p.(A20S) in the isoform ENSP00000379099) was added to data for the missense variants and this also showed evidence for association with the combined diseases (Fisher's *P*=0.0189; see Table 4).

rs4354668:A>C was found to influence the efficacy of lithium in a sample of Italian patients with BD.^46^ Case–case analysis showed that there is no association between the efficacy of lithium and rs4354668:A>C in our BD sample (*P*=0.74; OR=0.94).

rs4755404:C>G has been found to be associated with attempted suicide in a sample of psychiatric patients recruited in six Community Psychiatric Clinics in Ireland.^45^ Imputation of this SNP in GWAS data from the UCL1 BD cohort^52, 59^ was performed using the 1000 genome project data^56^ as a reference. These data were then analysed in a case versus case design with suicide attempt as the phenotype. In the UCL BD cohort there were 214 suicide attempters and 83 people recorded as never having attempted suicide. There was no evidence for association of rs4755404:C>G with attempted suicide in our sample (*P*=0.31).

## Discussion

The glutamate neurotransmission pathway has been implicated genetically and biologically in BD. The crucial role *SLC1A2* has in the uptake of the glutamate from the synaptic cleft and the previous genetic implication of the gene in BD suggests that this gene represents a potentially interesting gene to be studied in our BD and SZ cohorts.

The complexity of *SLC1A2* regulatory elements including alternative splicing of both the 5′- and 3′-UTR,^10, 11, 12, 13, 14^ protein isoforms with different N- and C-termini,^15, 16, 17^ enhancers, AU-rich elements, putative polyadenylation sites, exon-skipping splice variants and evidence of epigenetic regulation of human EAAT2^27, 28^ underline the importance of the fine tuning of this gene.

The importance of the fine regulation of this gene and the biphasic nature of BD makes a compelling argument for the existence of genetically determined pathological switch mechanisms, which may manifest themselves in the loss of control of gene expression. This led us to study not only the protein coding regions of the gene but also the putative regulatory regions including the 5′-UTR, the 3′-UTR, splicing sites and the putative promoter region. We identified several known and previously unreported variants. The variants that passed filtering were all genotyped in our BD and control cohorts.

*SLC1A2* has also been implicated in susceptibility to SZ and therefore the non-synonymous variants were also genotyped in our SZ cohort. The majority of the variants that were genotyped here were rare and none of the variants on their own were significantly associated with either BD or SZ. However, rs145827578:G>A has been found in three BD and two SZ patients and was absent in our control cohort. This variant causes a non-conservative amino acid change from p.(G6S) of the main isoform. Two other *SLC1A2* N-terminal non-synonymous variants in the main isoform were also detected in this study; case-only variant rs199599866:G>A (p.(R31Q)) was detected in one BD case and one SZ case; and ss825678893:C>T (p.(A7V)) was detected in one BD case and one control subject. These variants are extremely rare and they will have to be genotyped in substantially larger cohorts to understand the potential role of these variants in susceptibility to these diseases. However, combined analysis of the recurrent case-only non-synonymous SNPs in the main *SLC1A2* isoform in BD and SZ showed evidence for association with the two diseases together (*P*=0.0458).

The combined association findings for the recurrent case-only variants were strengthened when ss825678885:G>T, a 5′-UTR variant in one isoform, and a non-synonymous variant in a different isoform was included in the analysis (*P*=0.0189) and this may indicate that disruption of the expression of *SLC1A2* (discussed above) and/or the presence of missense variants in the N-terminal portion of the EAAT2 protein leads to increased susceptibility of developing BD and/or SZ. These tests of association have not been corrected for multiple testing and were performed *post-hoc* and therefore should be considered exploratory. However, it would be worthwhile investigating the effects of these rare variants on the function of EAAT2 to understand their contribution to the pathophysiology of disease in the carriers of these alleles.

While we have performed a systematic screen of the *SLC1A2* gene it is also possible that the N-terminal region of EAAT2 may harbour additional susceptibility variants for BD and SZ. The presence of new variants such as these will be revealed by large scale screening of this region either using a targeted approach or as part of whole exome or whole genome sequencing project.

We were not able to replicate the influence of rs4354668:A>C on the efficacy of lithium^46^ or the association of rs4755404:C>G with attempted suicide.^45^ These conflicting findings are not uncommon in the genetics of complex disease and may be due to differences in the genetic backgrounds of the samples or of sample size.

## Figures and Tables

**Table 1:** SNPs detected by HRM and sequencing analysis across the putative promoter region, 5′-UTR, exons, intron/exon junctions 3′-UTR regions of *SLC1A2* (GRCh37/hg19)

*SNP*	*Position on chr11*	*Variants*	*1000G MAF*	*Position in gene*
ss825678881:G>C	35441380	G>C	ND	Putative promoter
rs77619780:G>A	35441311	G>A	0.01	Putative promoter
rs11033118:C>T	35441254	C>T	0.01	Putative promoter
rs111885243:C>A	35440995	C>A	0.39	5′-UTR
rs4354668:A>C	35440976	A>C	0.59	5′-UTR
rs116392274:C>A	35440963	C>A	0.02	5′-UTR
ss825678882:A>G	35440927	A>G	ND	5′-UTR
ss825678884:G>A	35440635	G>A	ND	5′-UTR
ss825678883:C>G	35440563	C>G	ND	5′-UTR
rs145827578:G>A	35440498	G>A	ND	Exonic, non-synonymous
rs5791053:->T	35392657	->T	0.52	Intronic
rs1885345:T>C	35392601	T>C	0.87	5′-UTR
rs55643101:T>G	35392472	T>G	0.01	Intronic
ss825678885:G>T	35344202	G>T	ND	Exonic, non-synonymous/5′-UTR
rs56205617:G>A	35344107	G>A	0.03	Intron/exon junction
ss825678886:A>G	35344064	A>G	ND	Intronic
ss825678893:C>T	35339061	C>T	ND	Exonic, non-synonymous
rs199599866:G>A	35338989	G>A	ND	Exonic, non-synonymous
rs2273686:C>T	35338902	C>T	0.15	Intronic
rs2273687:C>G	35338893	C>G	0.21	Intronic
ss825678894:C>T	35336643	C>T	ND	Exonic, synonymous
rs56028027:A>G	35334032	A>G	0.25	Intronic
rs3895234:T>C	35327825	T>C	0.23	Intronic
rs116776036:C>T	35327804	C>T	0.01	Intronic
rs752949:G>A	35327748	G>A	0.23	Exonic, synonymous
rs2273689:T>C	35323200	T>C	0.59	Intronic
rs1042113:A>G	35308369	A>G	0.27	Exonic, synonymous
rs139804773:C>T	35302500	C>T	0.00	Exonic, synonymous
rs16927239:C>T	35302467	C>T	ND	Exonic, synonymous
ss825678896:G>T	35282548	G>T	ND	Intronic
ss825678895:C>T	35282452	C>T	ND	Exonic, non-synonymous
rs1570216:A>G	35282334	A>G	0.14	3′-UTR
rs10742339:T>A	35281100	T>A	ND	3′-UTR
rs147294857:C>T	35280227	C>T	0.01	3′-UTR
rs10768121:T>G	35279656	T>G	0.34	3′-UTR
ss825678897:A>G	35279202	A>G	ND	3′-UTR
rs12361171:A>T	35278333	A>T	0.34	3′-UTR
rs3812779:C>T	35276479	C>T	0.00	3′-UTR
rs1043101:T>C	35274829	T>C	0.34	3′-UTR
rs3088168:A>G	35273268	A>G	0.34	3′-UTR

Abbreviations: Chr, chromosome; HRM, high resolution melt; MAF, minor allele frequency; ND, not detected; SNP, single nucleotide polymorphism; UTR, untranslated region.

Variants, the base change indicated is on the negative strand.

**Table 2:** Polyphen 2 and SIFT prediction of coding non-synonymous variants in the different isoforms of *SLC1A2*

			*SLC1A2 isoforms*							
			*ENSP00000278379*		*ENSP00000379102*		*ENSP00000379099*		*ENSP00000476124*	
*Variant*	*aa changed*	*Programme*	*Prediction*	*Score*	*Prediction*	*Score*	*Prediction*	*Score*	*Prediction*	*Score*
rs145827578:G>A	p.(G6S)	Polyphen 2	Benign	0.439					Possibly Damaging	0.575
		SIFT	Tolerated	0.056						
ss825678885:G>T	p.(A20S)	Polyphen 2								
		SIFT								
ss825678893:C>T	p.(A7V)	Polyphen 2	Probably Damaging	0.993					Probably Damaging	0.996
		SIFT	Damaging	0.015						
rs199599866:G>A	p.(R31Q)	Polyphen 2	Benign	0.002	Benign	0.000	Benign	0.002	Benign	0.003
		SIFT	Tolerated	0.191	Tolerated	0.215	Tolerated	0.215		
ss825678895:C>T	p.(R572C)	Polyphen 2	Probably Damaging	0.978	Probably Damaging	0.978	Probably Damaging	0.978		
		SIFT	Damaging	0.003			Damaging	0.003		

Abbreviation: aa, amino acid.

aa change, the amino acid change indicated is the one in the ENSP00000278379 isoform for the variants rs145827578:G>A, ss825678893:C>T, rs199599866:G>A and ss825678895:C>T; the amino acid change indicated is the one in the uc021qfy.1 isoform for the variant ss825678885:G>T.

**Table 3:** Tests of association with *SLC1A2* variants in BD and SZ

	*Position on chr11*	*Position in gene*	*Change*	N	*Genotype count*		*MAF*	P	*OR (95%CI)*
ss825678881:G>C	35441380	Putative promoter	G>C	934	BD	0/3/931	0.0016	0.500	0.757 (0.1692–3.386)
				943	CTRL	0/4/939	0.0021		
rs77619780:G>A	35441311	Putative promoter	G>A	931	BD	1/19/911	0.0114	0.615	1.176 (0.6246–2.215)
				937	CTRL	1/16/920	0.0096		
rs11033118:C>T	35441254	Putative promoter	C>T	931	BD	0/20/911	0.0108	0.900	0.961 (0.5193–1.779)
				940	CTRL	1/19/920	0.0112		
ss825678882:A>G	35440927	5′-UTR	A>G	932	BD	0/3/929	0.0016	0.305	3.039 (0.3158–29.24)
				943	CTRL	0/1/942	0.0005		
ss825678884:G>A	35440635	5′-UTR	G>A	934	BD	0/2/932	0.001	0.492	2.018 (0.1828–22.28)
				942	CTRL	0/1/941	0.0005		
ss825678883:C>G	35440563	5′-UTR	C>G	934	BD	0/1/933	0.0005	0.493	NA
				941	CTRL	0/0/941	0		
rs145827578:G>A	35440498	Exonic p.(G6S)	G>A	1073	BD	0/3/1070	0.0013	0.149	NA
				631	SZ	0/2/629	0.0015	0.160	NA
				1704	BD+SZ	0/5/1699	0.0014	0.111	NA
				943	CTRL	0/0/943	0		
ss825678885:G>T	35344202	Exonic p.(A20S) 5′-UTR	G>T	1075	BD	0/2/1073	0.0009	0.281	NA
				626	SZ	0/0/626	0.0007	NA	NA
				1701	BD+SZ	0/2/1699	0.0005	0.415	NA
				944	CTRL	0/0/944	0		
rs56205617:G>A	35344107	Intronic	G>A	931	BD	1/53/877	0.0304	0.336	1.215 (0.8168–1.807)
				941	CTRL	1/44/896	0.025		
ss825678893:C>T	35339061	Exonic p.(A7V)	C>T	1072	BD	0/1/1071	0.0004	0.785	0.874 (0.05463–13.98)
				625	SZ	0/0/625	0	1.00	NA
				1697	BD+SZ	0/1/1696	0.0002	0.874	0.553 (0.0345–58.84)
				937	CTRL	0/1/936	0.0005		
rs199599866:G>A	35338989	Exonic p.(R31Q)	G>A	1065	BD	0/1/1064	0.0004	1.000	NA
				626	SZ	0/1/625	0.0007	0.398	NA
				1791	BD+SZ	0/2/1689	0.0005	0.415	NA
				940	CTRL	0/0/940	0		
ss825678895:C>T	35282452	Exonic p.(R572C)	C>T	1072	BD	0/2/1070	0.0009	0.737	0.879 (0.1236–6.243)
				630	SZ	0/0/630	0	0.358	NA
				1702	BD+SZ	0/2/1700	0.0005	0.448	0.554 (0.0779–53.935)
				942	CTRL	0/2/940	0.001		
rs147294857:C>T	35280227	3′-UTR	C>T	931	BD	0/34/897	0.0185	0.269	0.775 (0.4932–1.219)
				939	CTRL	0/44/895	0.0239		
ss825678897:A>G	35279202	3′-UTR	A>G	932	BD	0/3/929	0.0016	0.489	1.519 (0.2534–9.098)
				943	CTRL	0/2/941	0.001		
rs3812779:C>T	35276479	3′-UTR	C>T	930	BD	0/1/929	0.0005	0.491	NA
				944	CTRL	0/0/944	0		

Abbreviations: BD, bipolar disorder; chr, chromosome; CI, confidence interval; CTRL, control; MAF, minor allele frequency; NA, not applicable; OR, odds ratio; SZ, schizophrenia; UTR, untranslated region.

The genomic reference sequence used is GRCh37/hg19; change, the base change indicated is on the negative strand; genotype count, number of homozygotes for the minor allele/heterozygotes/homozygotes for the major allele; *N*, total number of research subjects; *P*, significance value for a two-tailed *χ*^2^-test and Fisher's exact test when cell frequencies were <10.

**Table 4:** Combined analysis of recurrent *SLC1A2* variants in BD and SZ

*Description of analysis*	*Variants included*	*Controls*	*BD and SZ*	P
Case-only non-synonymous	rs145827578:G>A (p.(G6S)) and rs199599866:G>A(p.(R31Q))	0/943	7/1697	0.0458
All case-only variants	rs145827578:G>A (p.(G6S)) rs199599866:G>A (p.(R31Q)) and ss825678885:G>T (p.(A20S)/5′-UTR)	0/944	9/1697	0.0189

Abbreviations: BD, bipolar disorder; SZ, schizophrenia.
